# Psychosocial and body image alterations associated with focal dystonia

**DOI:** 10.1007/s00702-026-03215-y

**Published:** 2026-06-29

**Authors:** Denise Mellace, Angelica Marfoli, Francesca Mameli, Angelica De Sandi, Carmelo Campo, Fabiana Ruggiero, Eleonora Zirone, Gabriella Santangelo, Valentina Tombola, Roberto Vimercati, Carlo Manzoni, Filippo Cogiamanian, Gaia Del Prete Ferrucci, Giordano D’Urso, Daniela Pia Rosaria Chieffo, Gabriella Pravettoni, Alberto Priori, Roberta Ferrucci

**Affiliations:** 1 Department of Oncology and Hemato-Oncology, University of Milan, Milan, Italy; 2 Foundation IRCCS Ca’ Granda Ospedale Maggiore Policlinico, Milan, Italy; 3 Department of Psychology, Luigi Vanvitelli University of Campania, Caserta, Italy; 4 University of Bath, Bath, UK; 5 Department of Psychiatry, Luigi Vanvitelli University of Campania Hospital, Naples, Italy; 6 Clinical Psychology Unit, Fondazione Policlinico Universitario Agostino Gemelli IRCCS, Rome, Italy; 7 Women, Children and Public Health Department, Catholic University of the Sacred Heart, Rome, Italy; 8 Applied Research Division for Cognitive and Psychological Science, European Institute of Oncology, IRCCS, Milan, Italy; 9 Neurological Clinic, ASST - Santi Paolo e Carlo, Department of Health Sciences, University of Milan, Milan, Italy; 10 Aldo Ravelli Center for Neurotechnology and Brain Therapeutics, Department of Health Sciences, University of Milan, Milan, Italy

**Keywords:** Focal dystonia, Body image, Depression, Anxiety, Quality of life (QoL)

## Abstract

Focal dystonia is a movement disorder characterized by involuntary muscle contractions resulting in abnormal postures and repetitive movements. Beyond motor impairment and physical disfigurement, dystonia is increasingly recognized as a network disorder with intrinsic non-motor symptoms, including body image concerns and psychological distress, which significantly affects quality of life (QoL). This study examined the psychosocial correlates of different forms of focal dystonia, focusing on altered body image, severity of anxiety and depression, and QoL in 19 patients with focal dystonia, 18 patients with hyperhidrosis (HH), and 13 patients with hemifacial spasm (HFS) as clinical control groups. Participants completed standardized tests including the Beck Depression Inventory-II for depression, the State-Trait Anxiety Inventory-Y2 for anxiety, the Body Uneasiness Test (BUT) for body image perception, and the 36-item Short Form Health Survey (SF-36) for QoL. Post-hoc comparisons revealed significant differences in both BUT and SF-36 scores between groups. Cervical dystonia (CD) patients had higher depersonalization symptoms than HH patients (BUT-D: 0.5 ± 0.7 vs. 0.1 ± 0.1; *p* = .027). CD patients also reported lower SF-36 Physical Role scores (55.6 ± 44.7) compared to blepharospasm (BPS) (87.5 ± 31.7), HH (95.8 ± 17.7), and HFS (65.4 ± 43.9) (*p* < .001). In contrast, BPS patients scored higher on SF-36 Emotional Role (90.0 ± 31.6) and SF-36 Social Functioning (89.9 ± 14.3) than CD (SF-36-ER: 70.2 ± 39.0, *p* < .001; SF-36-SF: 80.4 ± 25.1, *p* < .001). Depression and anxiety scores were below clinical thresholds and did not differ significantly between groups. These results support the view that non-motor symptoms are core features of focal dystonia, particularly in CD, and emphasize the need to address psychological and body image-related aspects in clinical management and future models of the disorder.

## Introduction

Dystonia is a network disorder characterized by the presence of sustained or intermittent muscle contractions determining abnormal, involuntary, repetitive movements and/or postures. These manifestations are often visible and may result in significant disfigurement and stigmatization (Bradnam et al. 2021; Camfield et al. 2002; Davidescu et al. 2021; Gündel et al. 2001; Paracka et al. 2020). Dystonic patients frequently experience embarrassment and dissatisfaction with their physical appearance and disabilities in everyday life, which can lead to social avoidance, impaired social interaction and isolation (Jahanshahi 2005; Lewis et al. 2008; Page et al. 2007). Prevalence and incidence data indicate an estimate between 2 and 50 cases per million for early-onset dystonia, and between 30 and 7320 cased per million for late-onset dystonia (Defazio 2010). Dystonias are classified along two axes: clinical characteristics and etiology. Clinical characteristics refer to the phenomenology of dystonia, including age of onset, association with other disorders, and body distribution. Based on distribution, patients are categorized into focal, segmental, multifocal, generalized, or hemidystonia (Fahn 1988). Focal dystonia, affecting isolated body regions, is ten times more common than the generalized form (Defazio and Gigante 2013). Common types of focal dystonia include cervical dystonia (CD) or torticollis (neck), blepharospasm when it affects eyes (BPS), oromandibular dystonia, arm dystonia, and laryngeal dystonia (De Carvalho Aguiar and Ozelius 2002; Pekmezovic et al. 2009). The second axis pertains to the etiology of dystonia covering anatomical changes and pathogenic disease patterns. Primary dystonias can be inherited or idiopathic, while secondary dystonias arise from other disease states or brain injury (Paracka et al. 2020). While primary focal dystonia has traditionally been considered as a movement disorder characterized primarily by painful involuntary postures and repetitive movements, recent studies suggest a high prevalence of psychological and psychiatric comorbidities (Santangelo et al. 2020), such as depressive, anxiety and obsessive-compulsive disorders (Fabbrini et al. 2010). Recent pathophysiological models conceptualize dystonia as a network disorder involving both motor and non-motor domains (Stamelou et al. 2012; Kuyper et al. 2011). Non-motor symptoms—including anxiety, depression, and alterations in body perception—are increasingly recognized as core clinical features of the disorder, rather than secondary reactions to motor disability. These symptoms can precede motor onset, their severity is not correlated with motor symptom severity, and they often persist despite improvement in motor function (Stamelou et al. 2012; Sugar and Pirio Richardson 2025). Importantly, affective disturbances appear to be more prevalent in dystonia than in other visible or socially stigmatizing conditions such as alopecia areata and hyperhidrosis (HH) (Gundel et al. 2003; Kuyper et al. 2011). These comorbidities are often associated with altered body perception, perfectionism and neuroticism in patients suffering from this condition (Ellement et al. 2021; Fabbrini et al. 2010; Lencer et al. 2009; Marfoli et al. 2024; Pérez-De-Heredia-Torres et al. 2021; Zurowski et al. 2013). In particular, evidence indicates that individuals with dystonia experience various social challenges stemming from distress over their body image, including social phobia, poor social interaction, social withdrawal, low self-esteem, and reduced quality of life (QoL) (Pekmezovic et al. 2009). Additionally, patients often face significant stigma related to their body image, adversely impacting their personal, social and occupational lives (Pérez-De-Heredia-Torres et al. 2021). For example, feeling unattractive and apologetic specifically about their appearance has been noted in patients with CD (Papathanasiou et al. 2001). Our recent study (Marfoli et al. 2024) found that patients with focal dystonia frequently experience symptoms related to body image concerns, such as body dysmorphic thoughts, dissatisfaction and feelings of embarrassment with their appearance, and social avoidance in interpersonal, sexual and occupational dimensions. Furthermore, research shows that patients with dystonia are at a higher risk for depression and anxiety symptoms and disorders, which may further diminish their quality of life (Fabbrini et al. 2010; Gündel et al. 2001; Lewis et al. 2008). In Fabbrini and colleagues’ work (2010), 89 patients with different forms of idiopathic isolated focal dystonia were compared to healthy controls, revealing that depressive disorders were significantly more prevalent in the dystonia group. Meta-analysis results also indicated that co-existing depressive symptoms substantially contribute to disability and decreased quality of life, with prevalence rates of 31.5% for CD, 29.2% for cranial dystonia, and 33.6% for mixed forms of focal dystonia (Medina Escobar et al. 2021). Thus, depression, alongside anxiety, appear to be a major factor influencing disability and quality of life in these individuals (Berardelli et al. 2015). At the same time, the social stigma associated with body changes and self-perceived physical defects may also play a role in the development and exacerbation of depression and anxiety in these patients (Medina Escobar et al. 2021; Moll et al. 2018). Visible motor disturbances affecting facial appearance can also be observed in other neurological conditions such as hemifacial spasm (HFS), which may similarly expose patients to psychosocial burden due to visible facial movements (Santangelo et al. 2020).

Despite several studies documenting the impact of depression and anxiety on the quality of life of individuals with dystonia (Blahak et al. 2008; Lencer et al. 2009; Smit et al. 2016; van den Dool et al. 2016), the subjective perception of body concept and disfigurement has received little attention in the past (Jahanshahi and Marsden 1990), although more recent work has begun to explore patient-reported experiences of body dissatisfaction, social stigma, and altered self-perception in focal dystonia (Marfoli et al. 2024; Ellement et al. 2021; Pérez-De-Heredia-Torres et al. 2021).

Building on this evidence, the present study aimed to investigate body image perception, anxiety and depressive symptoms, and health-related quality of life in patients with different forms of focal dystonia compared to two clinical control groups. Individuals with HH, a condition characterized by excessive sweating that can cause discomfort, embarrassment and psychological and social impairments, were included as a control representing a socially stigmatizing but non-neurological condition. In addition, patients with hemifacial spasm (HFS) were included as a second control group, representing a visible facial motor disorder with potential physical disfigurement. The inclusion of these two control groups allowed us to disentangle the effects of visible stigma from those related to disorder-specific mechanisms. Based on previous literature identifying non-motor symptoms as intrinsic features of dystonia (Kuyper et al. 2011; Stamelou et al. 2012; Sugar and Pirio Richardson 2025), we hypothesized that patients with dystonia would report poorer body image perception and lower quality of life compared to both control groups, reflecting alteration that extend beyond the psychosocial burden associated with visible or socially stigmatizing conditions. We have also explored potential associations between body perception alterations and measures of depression and anxiety.

## Materials and methods

The study population comprised 19 patients diagnosed with primary focal dystonia and recruited according to validated criteria in the outpatient clinic at the Movement Disorders clinic of the Foundation IRCCS Ca’ Granda Ospedale Maggiore Policlinico of Milan, Italy. The dystonic cohort included individuals with CD (*n* = 9) and BPS (*n* = 10). In addition, a group of 18 patients with primary focal HH (39.3 ± 9.0) and 13 patients with HFS (mean age ± SD, 65.6 ± 13.7) were enrolled for comparison. The HH group was included as a clinical control group, since both dystonia and HH are visible and potentially stigmatizing conditions treated within the same clinical setting. HH patients often experience embarrassment, social withdrawal, and reduced self-esteem related to their appearance (Parashar et al. 2023), despite the absence of neurological network dysfunction. The HFS group was included as a neurological control condition characterized by involuntary facial contractions that may be physically disfiguring and socially noticeable yet differ from dystonia in their underlying pathophysiology. This allowed comparison with a disorder presenting visible motor manifestations but not belonging to dystonia spectrum. These comparisons therefore allowed us to investigate whether the greater psychological burden and altered body image observed in dystonia reflect intrinsic, disorder-related mechanisms rather than a generic response to having a visible condition. Secondary forms of dystonia associated with other neurological abnormalities, except tremor resulting from dystonia, were excluded. Participants with major psychiatric disorders potentially accounting for psychopathological symptoms not causally related to dystonia were excluded using a clinical structured interview according to DSM-5 criteria (SCID-5-RV) (DSM-V, 2022; First et al. 2015). Demographic and clinical data, including age, gender, educational level, and disease duration are reported in Table 1. All participants were asked to complete the Beck Depression Inventory II (BDI-II) (Beck et al. 1961) to assess self-reported levels of depression and State-Trait Anxiety Inventory (STAI-Y2) (Iwasa et al. 1970) to determine the presence and the severity of anxiety symptoms. The 36-Item Short Form Health Survey (SF-36) (Ware et al. 1998; Ware and Sherbourne 1992) was also administered to evaluate patients’ quality of life and general health, and the Body Uneasiness Test (BUT) (Cuzzolaro et al. 2006) to investigate body image perception and dissatisfaction.

All the patients agreed to participate in this study. The experimental procedure was approved by the local institutional review board (Foundation IRCCS Ca’ Granda Ethics Committee) and was conducted in accordance with the Declaration of Helsinki. All patients were informed of the purpose of the study, and they gave their informed consent before participating.

### Beck Depression Inventory- II (BDI-II)

The BDI-II (Beck et al. 1961) was performed to evaluate the presence and severity of depression. It is a self-report questionnaire consisting of 21 items covering affective and somatic symptoms of depression. Higher total scores indicate more severe depressive symptoms. The cut-offs are the following: up to13 represent minimal depression, 14–19 mild depression, 20–28 moderate depression, and scores above 29 indicate severe depression.

### State Trait Anxiety Inventory (STAI-Y)

The STAI (Iwasa et al. 1970) consists of 40 items, with 20 measuring state anxiety (STAI-Y1) and other 20 measuring trait anxiety (STAI-Y2). State anxiety reflects an individual’s emotional condition at a specific moment, whereas trait anxiety refers to a stable personality characteristic. Each item is rated on a scale from 1 to 4, indicating frequency (almost never, sometimes, often, always). The total score can range from 20 to 80, with scores categorized as follows: 71–80 indicating very high anxiety, 51–70 indicating medium-high anxiety, 31–50 indicating medium-low anxiety, and 20–30 indicating very low anxiety or none.

### 36-Item Short Form Health Survey (SF-36)

The SF-36 is an instrument composed of 36 questions assessing the quality of life (QoL) and the general state of health (Ware et al. 1998; Ware and Sherbourne 1992). It is a multi-item scale evaluating both physical and mental components of functioning on a scale of 0 (total health impairment) to 100 (total well-being state). It is comprised of eight subscales: (1) Physical Functioning (PF) – limitations in physical activities due to health problems; (2) Physical Role (PR) – problems with work or other daily activities as a result of physical health; (3) Bodily Pain (BP) – intensity of pain and its interference with normal work; (4) General Health (GH) – personal evaluations of general health; (5) Vitality (V) – energy level and fatigue; (6) Social Functioning (SF) – the impact of physical or emotional problems on social activities; (7) Emotional Role (ER) – problems with daily activities due to emotional issues; (8) Mental Health (MH) – general mood and psychological well-being. Lower scores in SF-36 subscales reflect worse health-related quality of life.

### Body Uneasiness Test (BUT)

The BUT (Cuzzolaro et al. 2006) was employed to assess body dissatisfaction as well as symptoms of depersonalization related to one’s body image. This self-administered questionnaire comprises 34 items, with responses rated on a Likert scale from 0 (never) to 5 (always). The test yields multiple scores, including a total score and a Global Severity Index (GSI), which indicates the severity of issues associated with body self-image. Additionally, the BUT includes five subscales: Weight Phobia (WP), Body Image Concerns (BIC), Compulsive Self-Monitoring (CSM), Avoidance (A), and Depersonalization (D). Higher scores in BUT subscales reflect worse body uneasiness.

### Statistical analysis

The majority of psychological outcomes proved to distribute Normally (i.e., skewness and kurtosis values >|1| and >|3|, respectively) (Mishra et al. 2019), except for BDI, BUT-BIC, BUT-A, BUT-D, SF-36-PF, SF-36-PR, SF-36-BP, SF-36-SF and SF-36-ER scores. Hence, STAI-Y2, SF-36-V, SF-36-MH, SF-36-GH, BUT-GSI, BUT-PST, BUT-PSDI, BUT-CSM scores were analyzed *via* linear models (i.e., assuming an underlying Normal distribution), and remaining outcomes *via* generalized linear models. More specifically, BDI, SF-36-PF, SF-36-PR, SF-36-BP, SF-36-SF and SF-36-ER scores were analyzed *via* Negative Binomial models (Green 2021), whilst BUT-BIC, BUT-A and BUT-D were analyzed *via* Gamma models (Ng and Cribbie 2017). Both the Negative Binomial and the Gamma models are suitable for right-skewed, overdispersed data – with the differences between the two models lying in (1) the Negative Binomial being suited for discrete, integer values and the Gamma for non-integer, continuous ones, as well as in (2) the former allowing zeroes as compared to the latter.

Given that some of the abovementioned variables were left-, and not right-skewed, some adjustments needed to be done. More specifically, left-skewed SF-36 scores were reversed by subtracting from the empirical maximum the actual value in order for them to be modeled by the Negative Binomial. BDI scores did not need to undergo such an expedient since they were already right-skewed. As to BUT scores – which were non-integer and thus needed to be modeled by the Gamma distribution –, no reversal was necessary given that they were already right-skewed. However, since the Gamma does not allowed zeroes, a constant *k* = 0.01 was added to BUT-BIC, BUT-A, and BUT-D scores in order for them to be validly modeled by the analysis.

Since groups were unmatched for age, education and disease duration both when addressing the four patient groups (age: *F* (3,21) = 21.7; *p*<.001; education: *F* (3,19) = 5.1; *p* = .009 disease duration: *F* (3, 24) = 3.5; *p* = .031) and when addressing dystonia vs. HH and HFS groups (age: *F* (2, 27) = 33.1, *p* <.001; education: *F* (2, 27) = 6.6, *p* = .005; disease duration: *F* (2, 26) = 3.6, *p* = .043), these variables were entered as covariates within all of the abovementioned models.

Within all of these models, Bonferroni-corrected post-hoc comparisons were run for significant terms. The models above were run both for the four patient groups separately and for the three merged groups (dystonia vs. HH and HFS).

Analyses were run *via* Jamovi 2.3 (The Jamovi Project, 2022), using the GAMLj3 module.

## Results

Tables 1 and 2 show patients’ psychometric scores and report model results.

**Table 1:** Patients’ background and clinical measures and results of the models

	Blepharospasm	Cervical dystonia	Hemifacial spasm	Hyperhidrosis	*p*	Post-hoc comparisons
*N*	10	9	13	18	–	–
Age (years)	66.0 ± 11.3 (48–79)	60.8 ± 11.7 (46–80)	65.6 ± 13.7 (29–81)	39.3 ± 9.0 (20–50)	**< 0.001** ^**a**^	HH < CD, HFS, BPS
Sex (male/female)	3/7	2/7	7/6	8/10	.425^b^	–
Education (years)	9.8 ± 3.9 (5–13)	12.9 ± 3.2 (8–17)	12.2 ± 2.9 (8–17)	14.4 ± 1.9 (13–17)	**0.009** ^**a**^	HH > BPS
Disease duration (months)	16.9 ± 8.5 (3–27)	12.7 ± 5.4 (4–21)	15.8 ± 12.2 (2–44)	23.6 ± 11.7 (1–40)	.031^a^	HH > CD
BDI	3.0 ± 2.4 (0–9)	5.2 ± 4.1 (0–11)	8.8 ± 8.0 (0–26)	3.9 ± 4.3 (0–12)	.092^d^	
STAI-Y2	28.0 ± 8.7 (20–42)	38.6 ± 11.5 (22–59)	36.6 ± 10.7 (22–54)	35.8 ± 10.5 (20–54)	.118^c^	–
SF-36-PF	89.0 ± 18.1 (45–100)	81.1 ± 15.4 (60–100)	79.2 ± 25.4 (25–100)	97.8 ± 4.3 (85–100)	.173^d^	–
SF-36-PR	87.5 ± 31.7 (0–100)	55.6 ± 44.7 (0–100)	65.4 ± 43.9 (0–100)	95.8 ± 17.7 (25–100)	**< 0.001** ^**d**^	BPS > CD, HFS; HFS > CD; HH > CD, HFS
SF-36-BP	74.1 ± 26.2 (41–100)	62.6 ± 23.7 (41–100)	71 ± 29.7 (10–100)	95.6 ± 8.9 (72–100)	.363^d^	–
SF-36-GH	72.5 ± 21.4 (25–97)	60.9 ± 14.6 (32–76)	61.9 ± 21.1 (30–100)	79.8 ± 14.2 (42–97)	.119^c^	–
SF-36-V	75.0 ± 14.9 (50–95)	58.9 ± 17.3 (40–85)	61.9 ± 21.5 (25–100)	67.8 ± 12.5 (45–85)	.122^c^	–
SF-36-SF	89.9 ± 14.3 (62–100)	80.4 ± 25.1 (25–100)	75.8 ± 23.1 (25–100)	84.6 ± 21.0 (37–100)	**< 0.001** ^**d**^	BPS > CD, HFS, HH; CD > HFS; HH > HFS
SF-36-ER	90.0 ± 31.6 (0–100)	70.2 ± 39.0 (0–100)	63.9 ± 39.6 (0–100)	85.2 ± 34.8 (0–100)	**< 0.001** ^**d**^	BPS > CD, HFS, HH; CD > HFS; HH > HFS
SF-36-MH	86.0 ± 14.1 (52–100)	72.9 ± 15.2 (48–96)	70.5 ± 20.9 (44–100)	73.8 ± 13.5 (44–92)	.177^c^	–
BUT-GSI	0.4 ± 0.4 (0–1.2)	0.7 ± 0.7 (0–2.1)	0.5 ± 0.4 (0–1.2)	0.6 ± 0.4 (0–1.4)	.318^c^	–
BUT-PST	8.8 ± 8.8 (0–25)	13.4 ± 10.9 (0–30)	9.7 ± 7.7 (0–22)	12.6 ± 7.1 (0–23)	.531^c^	-
BUT-PSDI	1.5 ± 0.7 (0–2.3)	1.4 ± 0.7 (0–2.4)	1.4 ± 0.6 (0–2.4)	1.4 ± 0.5 (0–2.2)	.967^c^	-
BUT-BIC	0.6 ± 0.6 (0–1.6)	0.9 ± 1.0 (0–2.9)	0.7 ± 0.6 (0–1.6)	0.7 ± 0.6 (0–2.1)	.701^e^	–
BUT-A	0.3 ± 0.4 (0–0.8)	0.5 ± 0.8 (0–2.7)	0.4 ± 0.6 (0–1.7)	0.1 ± 0.3 (0–1.2)	.144^e^	–
BUT-CSM	0.4 ± 0.5 (0–1.5)	0.7 ± 0.7 (0–2.2)	0.5 ± 0.5 (0–1.3)	0.7 ± 0.4 (0–1.7)	.795^c^	–
BUT-D	0.1 ± 0.2 (0–0.6)	0.5 ± 0.7 (0–1.8)	0.2 ± 0.3 (0–1)	0.1 ± 0.1 (0–0.4)	**0.027** ^**e**^	CD > HH

*HH* Hyperhidrosis, *CD* Cervical Dystonia, *HFS* Hemifacial spasm, *BPS* Blepharospasm, *BDI* Beck’s Depression Inventory, *STAI-Y2*  Trait Anxiety Inventory, *SF-36*  Short Form Health Survey Questionnaire, *PF* physical functioning, *PR* physical role, *BP* bodily pain, *GH* general health, *VT* Vitality, *SF* social functioning, *ER* emotional role, *MH* mental health, *BUT* Body Uneasiness Test, *GSI* Global Severity Index, *PST* Positive Symptom Total, *PSDI* Positive Symptom Distress Index, *BIC* Body Image Concerns, *A* Avoidance, *CSM* Compulsive Self-Monitoring, *D* Depersonalization

^a^Welch’s *F*-statistic

^b^χ^2^-statistic

^c^General linear model

^d^Generalized linear model underlying a Negative Binomial distribution

^e^Generalized linear model underlying a Gamma distribution

Age, education and disease duration were entered as covariates within these models. Bold values indicate statistically significant results (p < 0.05).

As to the comparisons between the four groups, significant differences were detected in both SF-36 and BUT scores. More specifically, with regard to the SF-36-PR, post-hoc comparisons revealed that BPS patients reported higher scores when compared to those with HFS and CD, reflecting fewer physical limitations and better physical role functioning. Moreover, patients with HH showed higher SF-36-PR scores than those with CD and HFS and patients with HFS reported higher scores compared to those with CD. Concerning SF-36-SF scores, patients with BPS had higher scores compared to those with CD, HFS and HH, reflecting better perceived social functioning; further, CD and HH patients reported higher scores on this subscale of the SF-36 than those with HFS. With respect to the SF-36-ER, BPS patients showed higher scores compared to those with CD, HFS and HH, reflecting fewer emotional constraints in daily activities; additionally, patients with CD or HH reported higher scores than HFS patients. Finally, with regard to the BUT-D, patients with CD had higher scores compared to those with HH, reflecting greater detachment from their own body image.

When comparing dystonia patients as a whole with the HH and HFS cohorts, patients with dystonia showed significantly lower scores on the SF-36-PF and SF-36-PR subscales when compared to the HH group. Nonetheless, subjects with dystonia showed significantly higher scores on the SF-36-PF and SF-36-PR subscales compared to the HFS group, suggesting fewer physical limitations and issues related with work or other daily activities resulting from their physical health. Statistically significant differences were also observed on the SF-36-SF and SF-36-ER subscales, with dystonia patients reporting higher scores compared to the HFS group, indicating better social functioning and fewer complications with daily activities due to emotional issues. Significantly higher sores were detected on the BUT-D subscale, reflecting greater body uneasiness-related depersonalization in the dystonia patients compared to those with HH.

No significant difference emerged in the STAI and BDI scores between groups.

**Table 2:** Patients’ background, clinical measures and results of the models

	Dystonia	Hemifacial spasm	Hyperhidrosis	*p*	Post-hoc comparisons
*N*	19	13	18	–	–
Age (years)	63.5 ± 11.5 (46–80)	65.6 ± 13.7 (29–81)	39.3 ± 9.0 (20–50)	**< 0.001** ^**a**^	HH < DYT, HFS
Sex (male/female)	5/14	7/6	8/10	.263^b^	–
Education (years)	11.3 ± 3.9 (5–17)	12.2 ± 2.9 (8–17)	14.4 ± 1.9 (13–17)	**0.005** ^**a**^	HH > DYT
Disease duration (months)	14.9 ± 7.4 (3–27)	15.8 ± 12.2 (2–44)	23.6 ± 11.7 (1–40)	**0.043** ^**a**^	HH > DYT
BDI	4.1 ± 3.5 (0–11)	8.8 ± 8.0 (0–26)	3.9 ± 4.3 (0–12)	.093^d^	–
STAI-Y2	33.0 ± 11.2 (20–59)	36.6 ± 10.7 (22–54)	35.8 ± 10.5 (20–54)	.601^c^	–
SF-36-PF	85.3 ± 16.9 (45–100)	79.2 ± 25.4 (25–100)	97.8 ± 4.3 (85–100)	**< 0.001** ^**d**^	HH > DYT, HFS; DYT > HFS
SF-36-PR	72.4 ± 40.7 (0–100)	65.4 ± 43.9 (0–100)	95.8 ± 17.7 (25–100)	**< 0.001** ^**d**^	HH > DYT, HFS; DYT > HFS
SF-36-BP	68.6 ± 25.1 (41–100)	71 ± 29.7 (10–100)	95.6 ± 8.9 (72–100)	.212^d^	–
SF-36-GH	67.0 ± 19.0 (25–97)	61.9 ± 21.1 (30–100)	79.8 ± 14.2 (42–97)	.096^c^	–
SF-36-V	67.4 ± 17.7 (40–95)	61.9 ± 21.5 (25–100)	67.8 ± 12.5 (45–85)	.299^c^	–
SF-36-SF	85.4 ± 20.2 (25–100)	75.8 ± 23.1 (25–100)	84.6 ± 21.0 (37–100)	**< 0.001** ^**d**^	HFS < DYT, HH
SF-36-ER	80.6 ± 35.8 (0–100)	63.9 ± 39.6 (0–100)	85.2 ± 34.7 (0–100)	**< 0.001** ^**d**^	HFS < DYT, HH
SF-36-MH	79.8 ± 15.7 (48–100)	70.5 ± 20.9 (44–100)	73.8 ± 13.5 (44–92)	.286^c^	–
BUT-GSI	0.6 ± 0.6 (0–2.1)	0.5 ± 0.4 (0–1.2)	0.6 ± 0.4 (0–1.4)	.275^c^	–
BUT-PST	11.0 ± 9.9 (0–30)	9.7 ± 7.7 (0–22)	12.6 ± 7.1 (0–23)	.433^c^	–
BUT-PSDI	1.4 ± 0.7 (0–2.4)	1.4 ± 0.6 (0–2.4)	1.4 ± 0.5 (0–2.2)	.989^c^	–
BUT-BIC	0.8 ± 0.8 (0–2.9)	0.7 ± 0.6 (0–1.6)	0.7 ± 0.6 (0–2.1)	.521^e^	-
BUT-A	0.4 ± 0.6 (0–2.7)	0.4 ± 0.6 (0–1.7)	0.1 ± 0.3 (0–1.2)	.065^e^	–
BUT-CSM	0.5 ± 0.6 (0–2.2)	0.5 ± 0.5 (0–1.3)	0.7 ± 0.4 (0–1.7)	.866^c^	–
**BUT-D**	0.3 ± 0.5 (0–1.8)	0.2 ± 0.3 (0–1)	0.1 ± 0.1 (0–0.4)	**0.042** ^**e**^	DYT > HH

*DYT* Dystonia, *HFS* Hemifacial spasm, *HH* Hyperhidrosis, *BDI* Beck’s Depression Inventory, *STAI-Y2*  Trait Anxiety Inventory, *SF-36*  Short Form Health Survey Questionnaire, *PF* physical functioning, *PR* physical role, *BP* bodily pain, *GH* general health, *VT* Vitality, *SF* social functioning, *ER* emotional role, *MH* mental health, *BUT* Body Uneasiness Test, *GSI* Global Severity Index, *PST* Positive Symptom Total, *PSDI* Positive Symptom Distress Index, *BIC* Body Image Concerns, *A* Avoidance, *CSM* Compulsive Self-Monitoring, *D* Depersonalization

^a^Welch’s *F*-statistic

^b^χ^2^-statistic

^c^General linear model

^d^Generalized linear model underlying a Negative Binomial distribution

^e^Generalized linear model underlying a Gamma distribution

Age, education and disease duration were entered as covariates within these models. Bold values indicate statistically significant results (p < 0.05).

## Discussion

This study explored the multifaceted psychological and social challenges associated with focal dystonia, comparing these patients to two clinical control groups: individuals with HH, representing a socially stigmatizing but non-neurological condition, and individuals with HFS, representing a visible facial motor disorder with potential physical disfigurement. This design allowed us to examine whether alterations in body image perception and quality of life observed in dystonia could be explained by visible stigma alone or might reflect disorder-specific mechanisms. Overall, our findings highlight the significant burdens faced by individuals with dystonia, which extend beyond the physical symptoms to encompass serious mental health and quality of life concerns. In addition to previous studies (Berardelli et al. 2015; Fabbrini et al. 2010; Medina Escobar et al. 2021; Moll et al. 2018) showing that patients with focal dystonia may have comorbid depression and anxiety disorders affecting their quality of life, the aim of the present study was to investigate whether patients with focal dystonia, in particular CD and BPS, report uneasiness in physical appearance and alterations in body image perception, including body image concerns, social avoidance caused by embarrassment toward one’s body appearance, compulsive self-monitoring, and detachment and estrangement feelings toward one’s own body (depersonalization symptoms). In addition to dystonia patients, we also assessed individuals with HH and with HFS to compare psychological and social aspects, including body image concerns, body satisfaction, altered perception of self, and social withdrawal. A further objective of this study was to evaluate differences in health-related quality of life between the groups. We hypothesized that individuals with dystonia would report poorer outcomes in these areas compared to individuals with HH and with HFS, not only because of the visible disfigurement and potentially stigmatizing nature of their symptoms, but also due to the intrinsic involvement of non-motor networks in dystonia (Kuyper et al. 2011; Stamelou et al. 2012; Sugar and Pirio Richardson 2025).

The results confirmed a significant difference between the groups. Notably, dystonia patients reported more severe depersonalization symptoms compared to those with HH, particularly in relation to body concept. Specifically, the BUT test revealed that patients with CD experienced greater body dissatisfaction and more pronounced feelings of depersonalization than their HH counterparts. These findings are consistent with recent literature highlighting that altered body perception and psychological symptoms are core features of dystonia, rather than secondary reactions to motor manifestations (Kuyper et al. 2011; Stamelou et al. 2012; Sugar and Pirio Richardson 2025). The stigma associated with these visible symptoms may further exacerbate negative self-perception and social withdrawal, reinforcing symptoms of depression and anxiety in these patients (Pérez-De-Heredia-Torres et al. 2021). Our study also corroborated previous research (Ben-Shlomo et al. 2002; Girach et al. 2019; Junker et al. 2021; Pekmezovic et al. 2009; Smit et al. 2016) indicating that patients with HH report higher quality of life scores in areas of physical functioning and interference with daily life compared to patients with dystonia. This suggests that individuals with dystonia, particularly CD, experience more significant challenges in these domains due to their physical symptoms. Patients with HFS showed even lower scores that dystonia patients in SF-36 domains related to physical functioning and role limitations, suggesting a more pronounced impact on everyday activities and perceived functional capacity. A similar pattern emerged in social participation and emotional well-being, where patients with HFS reported even lower scores than both dystonia and HH groups, likely reflecting the strong psychosocial impact of a disfiguring facial motor disturbance. Dystonia did not show the same degree of functional, social and emotional impairment in our cohort but was characterized by greater alterations in body perception in comparison with the HH group. This pattern may suggest that disturbances in bodily representation are more pronounced in dystonia than in a socially stigmatizing but non-neurological condition, supporting the idea that non-motor aspects of dystonia extend beyond the impact of stigma alone (Kuyper et al. 2011; Stamelou et al. 2012; Sugar and Pirio Richardson 2025).

In comparing the specific forms of focal dystonia—CD and BPS—with HH, differences emerged regarding both quality of life and body image perception. Patients with BPS and HH scored higher on physical role, social functioning, and emotional well-being subscales of the SF-36 compared to those with CD. This trend is consistent with previous evidence indicating that patients with BPS may experience comparatively less impairment in quality of life than those with CD (Pekmezovic et al. 2009). Overall, these findings indicate subtype-specific variation in quality of life and psychological outcomes, highlighting the importance of considering the heterogeneity of non-motor features when assessing the psychological impact of dystonia. Furthermore, our analysis highlighted that patients with CD experience the most significant psychological distress related to body image, as evidenced by their more frequent and severe depersonalization symptoms. This suggests that CD may profoundly affect one’s self-perception, with both visible stigma and perceived or internalized stigma likely contributing to the psychological burden (Gündel et al. 2001; Rinnerthaler et al. 2006; Monaghan et al. 2020). Indeed, previous research has shown that social anxiety in CD is not directly correlated with motor symptom severity (Gündel et al. 2001), suggesting that self-stigmatization and perceived negative evaluation contribute substantially to patients’ distress. Consistent with this, recent research by O’Connor and colleagues (2023) found high levels of distress in CD patients, with 51% experiencing anxiety and 28.8% experiencing depression. The stigma surrounding CD may contribute to feelings of unattractiveness, self-consciousness, and reduced self-esteem, particularly in public settings where the condition’s visible symptoms are more likely to attract attention (Papathanasiou et al. 2001). Additionally, data shows that people with cranial and cervical dystonia often face public misunderstandings about whether they are responsible for their condition (Rinnerthaler et al. 2006). Although symptoms of anxiety and depression have been frequently reported in patients with focal dystonia (Fabbrini et al. 2010; Gündel et al. 2001; Lewis et al. 2008), in our cohort the BDI and STAI-Y2 scores were below the clinical threshold, with no significant differences between groups, suggesting that in this sample the psychological distress is mild and that altered body perception and quality of life better reflect non-motor impact.

In conclusion, our study provides further evidence that focal dystonia, particularly CD, is associated with a significantly poorer psychological and social quality of life. While visible and socially stigmatized symptoms contribute to this burden, our findings suggest that alterations in body perception is an intrinsic feature of dystonia rather than a mere consequence of stigma. These findings underscore the need for targeted psychological support for patients suffering from this movement disorder, particularly those with CD, to address issues related to body image, self-esteem, and social integration.

While this study offers valuable insights, it is not without limitations. The relatively modest sample size may limit the generalizability of the findings, underscoring the need for future research involving larger cohorts to validate these results. Additionally, future studies should examine the long-term effects of dystonia on mental health and quality of life, as well as assess the effectiveness of targeted psychological interventions.

These findings underscore that non-motor symptoms constitute intrinsic aspects of dystonia, warranting equivalent consideration as motor features in both clinical practice and future pathophysiological models of the disorder. They also emphasize the importance of adopting a multidisciplinary approach to managing dystonia that goes beyond physical treatment alone. Incorporating psychological support into routine care is essential to address the mental health challenges faced by patients. Therapeutic interventions such as cognitive-behavioral therapy and social skills training may be particularly helpful in addressing body image concerns and improving social interactions. Moreover, increasing awareness and education about dystonia is crucial to reducing stigma. The establishment of support groups and community programs would offer valuable spaces for patients to exchange experiences and coping strategies, fostering a sense of community and helping to mitigate feelings of isolation.

## Declarations

### Data availability

Datasets related to the present study are available upon reasonable request from interested researchers.
